# Comparative effects of the herbal constituent parthenolide (Feverfew) on lipopolysaccharide-induced inflammatory gene expression in murine spleen and liver

**DOI:** 10.1186/1476-9255-2-6

**Published:** 2005-06-29

**Authors:** Alexa T Smolinski, James J Pestka

**Affiliations:** 1 Department of Food Science and Human Nutrition, Michigan State University, East Lansing, Michigan, USA; 2 Institute for Environmental Toxicology, Michigan State University, East Lansing, Michigan, USA; 3 Department of Microbiology and Molecular Genetics, Michigan State University, East Lansing, Michigan, USA

## Abstract

**Background:**

Parthenolide, a major sesquiterpene lactone present in extracts of the herb Feverfew, has been investigated for its inhibitory effects on mediators of inflammation, including the proinflammatory cytokines. Although parthenolide's anti-inflammatory effects have been investigated in vitro, little in vivo data are available. Moreover, the molecular mechanisms for these inhibitory effects are not fully understood. The objective of this study was to test the hypothesis that parthenolide suppresses lipopolysaccharide (LPS)-induced serum (interleukin) IL-6, tumor necrosis factor (TNF)-α, IL-1β and cyclooxygenase (COX)-2 expression in mice as indicated by reduced splenic and liver mRNA levels.

**Methods:**

Mice were co-treated i.p. with LPS (1 mg/kg bw) and parthenolide (5 mg/kg bw) and blood, spleen and liver collected. Serum was analyzed for IL-6, TNF-α and IL-1β by ELISA. Total RNA was extracted from spleen and liver, and real-time RT-PCR was used to determine relative mRNA expression of IL-1β, IL-6, TNF-α and COX-2.

**Results:**

LPS induced increases in serum IL-6 and TNF-α concentrations with only IL-6 being suppressed in parthenolide-treated mice. Induction of IL-6 mRNA was reduced, TNF-α and COX-2 mRNAs unchanged, and IL-1β mRNA increased in spleens of parthenolide plus LPS co-treated animals compared to LPS-only. No significant differences were observed in inflammatory gene expression between these two groups in liver samples. Overall, mRNA expression of each proinflammatory gene was much higher in spleen when compared to liver.

**Conclusion:**

In summary, only one gene, IL-6, was modestly suppressed by parthenolide co-exposure which contrasts with many in vitro studies suggesting anti-inflammatory effects of this compound. Also, LPS evoked greater effects in spleen than liver on expression of proinflammatory genes. Further study of the effects of parthenolide and other herbal constituents on inflammatory gene expression using model animal systems as described here are critical to evaluating efficacy of such supplements as well as elucidating their mechanisms of action.

## Background

Parthenolide, the major sesquiterpene lactone derived from the feverfew extract (*Tanacetum parthenium)*, has been studied for its inhibitory effects on inflammation in cell culture and, to a limited extent, in live animals. This constituent has been shown to attenuate a variety of inflammatory endpoints [1-12]. Recent attention has turned to the determination of the molecular mechanisms by which parthenolide imparts its effects on inflammatory responses.

Investigations of the anti-inflammatory properties of parthenolide, and feverfew have focused on suppression of primary inflammatory endpoints such as platelet aggregation [1] and carrageenan-induced mouse [2] and rat [3] paw edema. Additional studies have evaluated parthenolide's inhibitory effect on inflammatory mediators including activity and expression of cyclooxygenase (COX) [4,5], generation of prostaglandins [6,7], and leukotrienes (LT) [4] and expression of proinflammatory cytokines [5,8]. Most recently, the compound was found to inhibit activation of transcription factor nuclear factor (NF)-κB [9-12].

Previous research in our laboratory focused on the inhibitory effects of parthenolide on lipopolysaccharide (LPS)-induced proinflammatory cytokine production in the supernatant of murine cell culture and sera of animals [13]. The data showed that parthenolide impairs LPS-induced tumor necrosis factor (TNF)-α and interleukin (IL)-6 upregulation in culture and in sera of animals when parthenolide was administered via i.p. injection.

Although protein levels of LPS-induced proinflammatory cytokines are reportedly reduced by parthenolide treatment, there are limited data evaluating the effect of parthenolide on mRNA expression of these cytokines. Hwang et al. [5] showed that parthenolide suppresses LPS-induced steady state levels of TNF-α and IL-1β mRNA in cell culture. Parthenolide had no inhibitory effect on IL-6 mRNA levels in LPS-stimulated macrophages, but did attenuate IL-12 p40 and p35 mRNA expression [14] as well as the chemokine IL-8 in cultured human respiratory epithelium [15].

Parthenolide's effects on specific cytokine gene expression have been documented in vitro, but, to our knowledge, few data are available regarding effects on mRNA expression of cytokines or other inflammatory genes such as COX-2 in vivo. This is an important consideration because absorption, distribution and metabolism of this compound will likely impact how it affects inflammation in the host. The objective of this study was to test the hypothesis that parthenolide-induced suppression of serum LPS-induced IL-6 and TNF-α correlate with reduced mRNA levels for these genes, and other related proinflammatory genes, in the spleen and liver which are tissues well-known to express IL1β, IL-6, TNF-α and COX-2. Additionally, we sought to determine whether differences in expression levels of each gene existed between the spleen and liver. These organs contain macrophages and other cell types capable of responding to LPS and other inflammatory stimuli.

## Methods

### Chemicals

All chemicals were purchased from Sigma Chemical Co. (St. Louis, MO) unless otherwise noted. Parthenolide (Calbiochem, San Diego, CA) was dissolved in tissue culture grade dimethyl sulfoxide (DMSO). Lipopolysaccharide (LPS) from *Salmonella typhimurium *[1.5 EU/ng LPS; Stimulation index (SI) 3.6 @15.6 μg/ml LPS] was dissolved in endotoxin-free tissue culture grade water.

### Experimental design

All animal handling was conducted in accordance with guidelines established by the National Institutes of Health. Experiments were designed to minimize the numbers of animals used. Female B6C3F1 mice (8–10 weeks) were obtained from Charles River (Portage, MI). Animals were housed 3 or 4 per cage with a 12 h light/dark cycle, provided standard rodent chow and water *ad libitum*, and acclimated to their environment at least one week before the start of experiments.

Chow and water were removed from cages one hour prior to the start of each experiment. Mice were co-treated with 5 mg/kg, i.p. (in 50 μl DMSO) parthenolide and 1 mg/kg, i.p. LPS (in 100 μl water). This parthenolide dose was selected based on solubility limitations and because it was the lowest dose to show consistent inhibition of IL-6 and TNF-α elevation in four preliminary studies using doses at 0.05, 0.5, 1, 5 and 10 mg/kg. Vehicle-treated mice received 50 μl DMSO, i.p. and 100 μl water, i.p. Parthenolide control animals received parthenolide 5 mg/kg, i.p. and 100 μl water, i.p. After 90 minutes, blood was collected by retro-orbital bleeding under methoxyflurane anesthesia. Animals were then immediately euthanized by cervical dislocation and spleen and liver were collected. The time interval was chosen based on preliminary studies of LPS induction in expression of the four target genes. This euthanasia method was chosen to minimize artifactual immunologic effects [16] and was approved by MSU All University Committee on Animal Research and Care.

### Serum IL-6, TNF-α, and IL-1β determination by ELISA

Blood was allowed to clot overnight at 4°C. Serum was analyzed for IL-6, TNF-α and IL-1β by ELISA. IL-6 analysis was performed using purified and biotin-conjugated rat anti-mouse IL-6 antibodies from PharMingen (San Diego, CA) as described previously [13]. Streptavidin-peroxidase (Sigma) and 3,3',5,5'-tetramethylbenzidine (TMB, Fluka, Ronkonkoma, NY) were used for detection. Absorbance was read at 450 nm using a Vmax™ Kinetic Microplate Reader (Molecular Devices, Menlo Park, CA). For TNF-α analysis the OptEIA Set: Mouse TNF-α (Mono/Poly) kit was employed (PharMingen). For IL-1β analysis, a DuoSet^® ^ELISA (R&D Systems, Minneapolis, MN) was used. The sensitivity of all three ELISAs was 20 pg/ml.

### Total RNA extraction from spleen and liver

Spleens and livers were cut into small pieces and placed into TRIzol^® ^Reagent (Invitrogen Life Technologies, Carlsburg, CA). Samples were homogenized for 30 seconds at setting 8 using a Polytron^® ^Homogenizer (Brinkmann, Westbury, NY) and RNA extractions were completed according to manufacturer's instructions. Total RNA was quantified at 260 nm using a GeneQuant RNA/DNA Calculator (Pharmacia Biotech, Cambridge, England).

### mRNA quantification from spleen and liver

Relative IL-6, TNF-α, IL-1β and COX-2 mRNA levels were determined according to manufacturer's instructions using TaqMan ^® ^real-time reverse transcription (RT)- polymerase chain reaction (PCR), ABI Prism^® ^7700 Sequence Detection System (Applied Biosystems, Foster City, CA) and Applied Biosystems reagents unless indicated otherwise. The RT-PCR reaction was carried out in a total reaction volume of 25 μl containing: 1) RNase-free water (Sigma) to 25 μl; 2) 12.5 μl TaqMan^® ^One-Step RT-PCR Master Mix Reagent; 3) 1.25 μl either IL-6, TNF-α or IL-1β Pre-Developed Assay Reagent (primer and probe sets); 4) 1.25 μl 18S rRNA Pre-Developed Assay Reagent; 5) 50 ng total RNA in RNase-free water and 6) 0.63 μl MultiScribe and RNase Inhibitor Mix. COX-2 mRNA was similarly analyzed using forward 5'-CAGAAC CGCATT GCCTCTG-3' and reverse 3'-AGCTGTACTCCTGGTCTTCAATGTT-5' primers (900 nM each) (Michigan State University Genomics Facility, East Lansing, MI) and probe 6FAM-CAACACACTCTATCACTGGCACCCCCTG-TAMRA (250 nM) designed using Primer Express™ software (Applera Corporation, Norwalk, CT). All samples were multiplexed with 18S rRNA which served as an endogenous reference for cytokine mRNA normalization. All samples were assayed in duplicate and serial dilutions of standard (total RNA from LPS-treated mouse spleen) in triplicate. No template control and no RT negative control reactions were also performed. Reaction conditions were: 48°C for 30 min; 95°C for 10 min; and 40 cycles of 95°C for 10 seconds and 60°C for 1 min.

### Statistics

All statistical analyses were performed using SigmaStat Statistical Analysis Software (Jandel Scientific, San Rafael, CA). For comparison of two groups, a Student's *t*-test was used. For comparisons of multiple groups using parametric data, one-way analysis of variance (ANOVA) using Student-Newman-Keuls Method for all pairwise multiple comparisons was performed.

## Results

### Parthenolide co-treatment in vivo inhibits LPS-induced IL-6 protein production in serum

**Figure 1:**
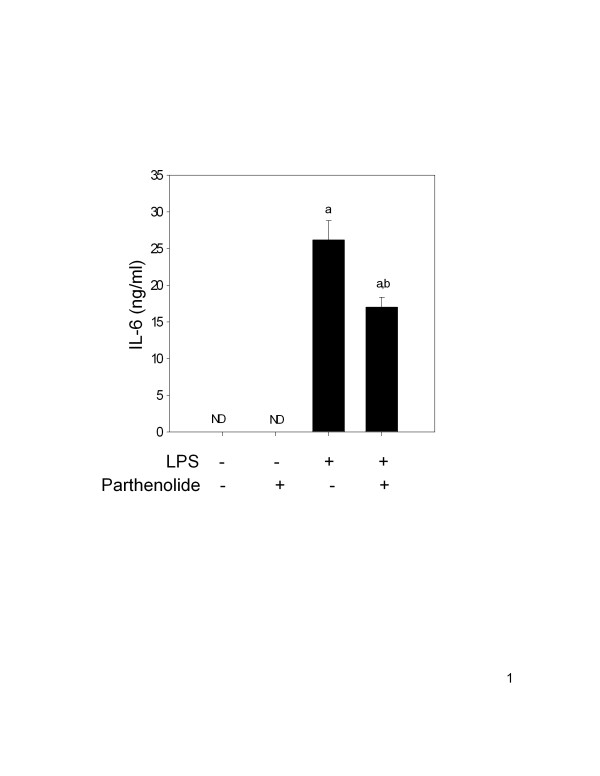
IL-6 protein production in sera following parthenolide and LPS co-treatment. Female B6C3F1 mice were co-treated with parthenolide (5 mg/kg, i.p.) or 50 μl DMSO and LPS (1 mg/kg, i.p.) or 100 μl water. After 90 minutes, blood was collected and serum analyzed for IL-6 by ELISA. The letter (a) indicates a significant difference compared to vehicle and parthenolide controls; (b) indicates a significant difference compared to LPS. Data are mean ± SEM (n = 16, controls n = 4), and is a combination of 4 separate experiments.

In order to determine the systemic effect of parthenolide co-treatment on LPS-induced IL-6 production, mice were treated with parthenolide (5 mg/kg, i.p.) and LPS (1 mg/kg, i.p.) for 90 minutes. Blood was collected and serum analyzed for IL-6. Animals treated with LPS alone produced 26 ± 2.6 ng/ml of IL-6 (Fig. 1). Serum concentrations of IL-6 were not detectable in vehicle and parthenolide control animals. Co-treatment with parthenolide caused a 35 percent reduction in LPS-induced IL-6 production compared to animals treated with LPS alone (P < 0.05).

### Parthenolide co-treatment in vivo impairs LPS-induced IL-6 mRNA expression in spleen but not liver

**Figure 2:**
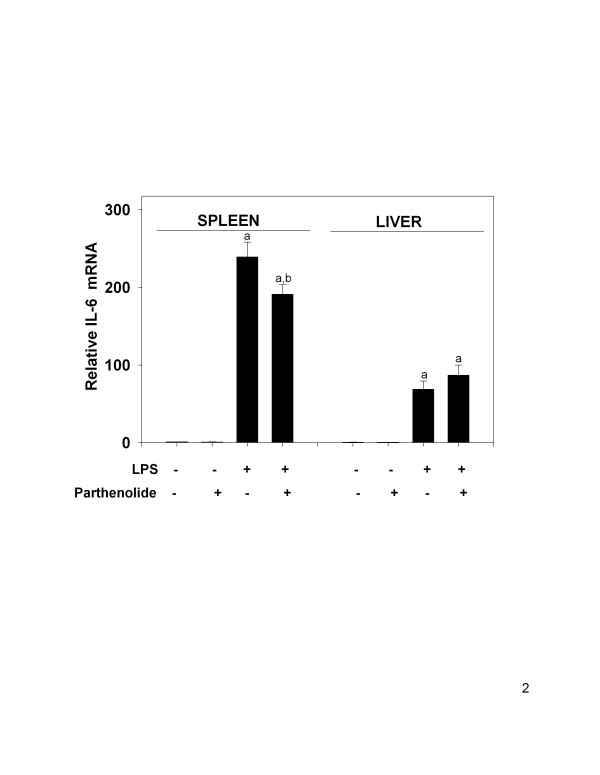
IL-6 mRNA expression levels in spleen and liver following parthenolide and LPS co-treatment. Female B6C3F1 mice were co-treated with parthenolide (5 mg/kg, i.p.) or 50 μl DMSO and LPS (1 mg/kg, i.p.) or 100 μl water. Spleen and liver were collected after 90 minutes and total RNA was extracted and subjected to real-time, one-step RT-PCR using TaqMan primers and probes. IL-6 mRNA levels were normalized using 18S rRNA and related to spleen control values. (a) indicates a significant difference compared to vehicle and parthenolide controls; (b) indicates a significant difference compared to LPS. Data are mean ± SEM (n = 16, controls n = 4), and is a combination of 4 separate experiments.

Relative IL-6 mRNA expression in the spleen and liver of co-treated animals was also determined by real-time RT-PCR. IL-6 mRNA expression was significantly induced in spleen 239 ± 19-fold spleen control) and liver (117 ± 18-fold liver control) (Fig. 2). IL-6 expression in vehicle and parthenolide control animals was negligible in both spleen and liver samples. Splenic IL-6 mRNA levels of parthenolide and LPS co-treated animals (191 ± 12-fold) was 20 percent less than compared to spleens from LPS-only treated animals (p < 0.05), but not significantly different in liver (P < 0.05). Overall, IL-6 mRNA expression in spleen was 2.8-fold higher than the liver in LPS-treated animals, and was 1.4-fold higher in the spleen of animals receiving LPS plus parthenolide co-treatment compared to that of the liver.

### Parthenolide co-treatment in vivo does not inhibit LPS-induced TNF-α protein production in serum

**Figure 3:**
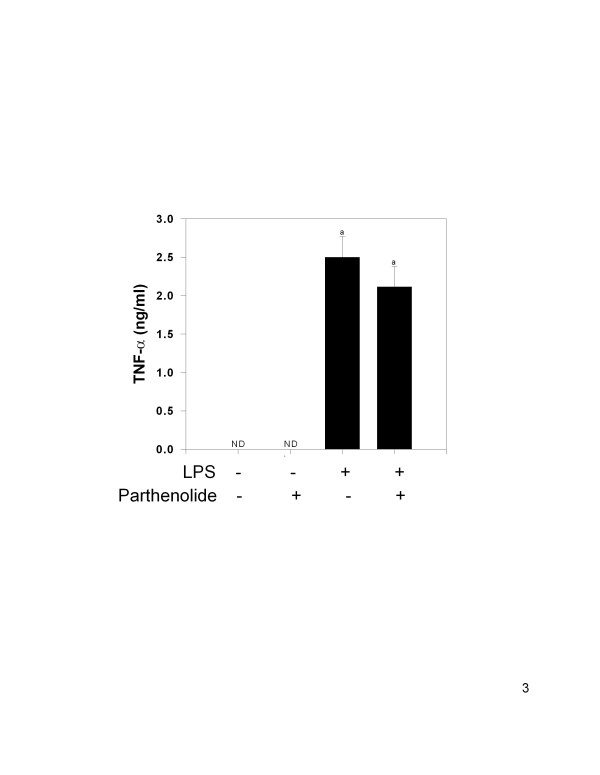
TNF-α protein production in sera following parthenolide and LPS co-treatment. Mice were treated and sera analyzed for TNF-α as described in Fig. 1 legend. The letter (a) indicates a significant difference compared to vehicle and parthenolide controls. Data are mean ± SEM (n = 16, controls n = 4), and is a combination of 4 separate experiments.

LPS-treated animals exhibited significantly increased TNF-α concentration (2.50 ± 0.27 ng/ml) in sera compared to both vehicle and parthenolide control animals (Fig. 3). TNF-α was not detectable in either control group. TNF-α concentrations in animals co-treated with parthenolide plus LPS (2.11 ± 0.26 ng/ml) were not significantly different from LPS-only treated animals (P < 0.05) although there was a downward trend.

### Parthenolide co-treatment in vivo does not affect LPS-induced TNF-α mRNA in spleen and liver

**Figure 4:**
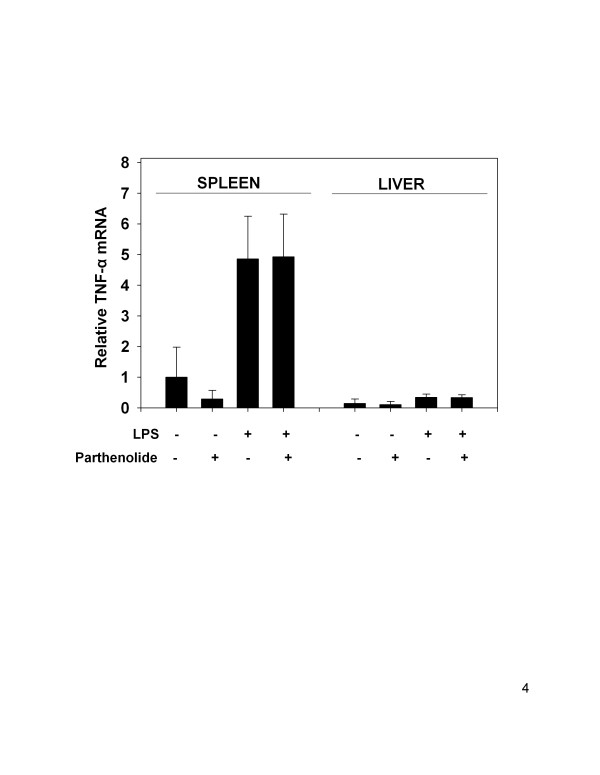
TNF-α mRNA expression levels in spleen and liver following parthenolide and LPS co-treatment. Mice were treated and analyzed for TNF-α mRNA described in Fig. 2 legend. TNF-α mRNA levels were normalized using 18S rRNA and related to spleen control values. Data are mean ± SEM (n = 16, controls n = 4), and is a combination of 4 separate experiments.

TNF-α mRNA was also increased in the spleen (4.84 ± 1.4-fold) and liver (2.33 ± 0.71-fold) of LPS-treated animals over controls. In both the spleen and liver there were no differences in TNF-α mRNA expression between LPS-treated and LPS plus parthenolide-treated mice (Fig. 4) (P < 0.05). In fact, there was no statistical differences among any of the groups evaluated in this study (P < 0.05). LPS-induced splenic TNF-α mRNA levels were considerably higher (14-fold) than those in the liver.

### Parthenolide co-treatment in vivo elevates LPS-induced IL-1β mRNA in spleen but not liver

**Figure 5:**
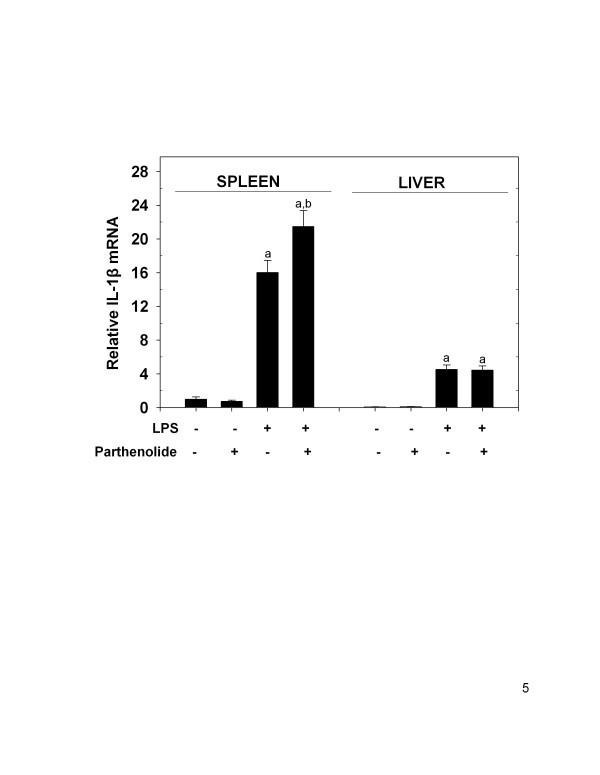
IL-1β mRNA expression levels in spleen and liver following parthenolide and LPS co-treatment. Mice were treated and analyzed for IL-1β mRNA as described in Fig. 2 legend. The letter (a) indicates a significant difference compared to vehicle and parthenolide controls; (b) indicates a significant difference compared to LPS. Data are mean ± SEM (n = 16, controls n = 4), and is a combination of 4 separate experiments.

Serum IL-1β was not detectable in any of the groups tested. However, IL-1β mRNA expression was significantly elevated in spleen (16.01 ± 1.45-fold) and liver (62.2 ± 7.43-fold) of LPS-only treated animals in comparison to vehicle and parthenolide control animals (Fig. 5). The level of IL-1β mRNA in spleen and liver of vehicle and parthenolide control animals was negligible. In the spleens of co-treated animals, there was a significant (p < 0.05) increase in IL-1β mRNA (21.48 ± 6.91-fold) compared to LPS-only treated animals. IL-1β mRNA expression was 3.2-fold higher in spleen of LPS-only treated animals, and 4.5-fold higher in LPS plus parthenolide co-treated animals, when compared to expression levels of the liver.

### Parthenolide co-treatment in vivo does not affect LPS-induced COX-2 mRNA in spleen and liver

**Figure 6:**
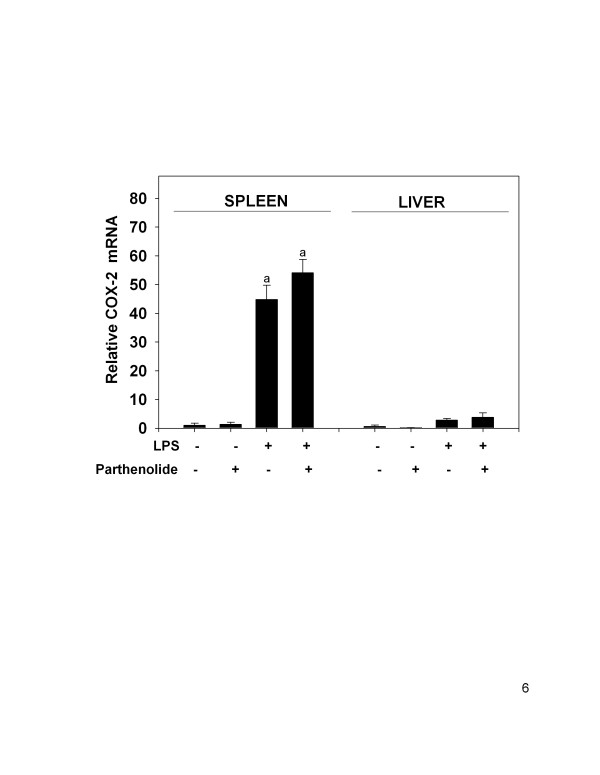
COX-2 mRNA expression levels in spleen and liver following parthenolide and LPS co-treatment. Mice were treated and COX-2 mRNAs analyzed as described in Fig. 2 legend. The letter (a) indicates a significant difference compared to vehicle and parthenolide controls. Data are mean ± SEM (n = 16, controls n = 4), and is a combination of 4 separate experiments.

Relative COX-2 mRNA expression was assessed in the spleen and liver of parthenolide plus LPS co-treated animals. COX-2 mRNA expression was markedly induced in the spleen of LPS treated animals (44.8 ± 5.0-fold over control) and increased to a lesser extent in liver (4.6 ± 0.9) (Fig. 6). In both spleen and liver samples there were no significant differences in COX-2 mRNA expression between LPS-treated and LPS plus parthenolide-treated mice (P < 0.05). Overall, COX-2 mRNA expression levels were 15.6- and 14.2-fold higher in spleen of LPS-treated mice and parthenolide plus LPS-treated mice, respectively, when compared to expression levels observed in liver.

## Discussion

Parthenolide has been demonstrated to inhibit inflammatory gene expression in vitro (4–8, 13). The results of this study are important because it is the first, to our knowledge, to evaluate parthenolide's effect on inflammatory gene expression in two primary sites of the LPS response – the spleen and the liver. The data indicate that protein concentrations in serum followed a similar trend to splenic mRNA accumulation for IL-6 in LPS and parthenolide/LPS co-treated mice. However protein and splenic mRNA levels were not consistent with liver samples. The mRNA levels of each inflammation-related gene in the liver was not changed, irrespective of parthenolide co-treatment, when compared to LPS alone.

There is a marked contrast between the robust attenuation by parthenolide of proinflammatory gene expression reported in vitro (4–12, 15) and the small responses reported in animals here and previously (13). It is possible that in the whole animal the kinetics absorption, metabolism and distribution and clearance of parthenolide preclude sufficient contact time in target immune tissue to evoke potent attenuation of LPS response. These factors must be considered when attempting to extrapolate an in vitro effect of an herbal compound to the in vivo situation.

Serum IL-6, but not TNF-α, was significantly reduced following co-treatment with parthenolide (5 mg/kg, i.p.) and LPS (1 mg/kg, i.p.) compared to animals receiving LPS alone. Similarly, IL-6 mRNA concentration in the spleens of co-treated animals was significantly reduced, whereas splenic TNF-α mRNA, and COX-2, were not changed as compared to LPS- treated animals. In contrast, the level of IL-1β mRNA in the spleen was significantly elevated in co-treated mice but no effects were observed in the liver. Serum IL-1β were also evaluated, but levels were below the limit of detection in all treatment groups (data not shown). Absence of serum IL-1β might result from delay in translation/secretion of the protein after transcription, receptor binding or degradation. Regardless of the cause, comparisons could not be made between protein production and mRNA expression for IL-1β in this study.

The observed mRNA expression levels of the proinflammatory cytokines and COX-2 might be higher in spleen than liver because of inherent phagocytic capacities of the macrophage cell populations within each organ. In the spleen, macrophages play key roles in phagocytosis, especially of nonopsonized particles, whereas the macrophage of the liver, Kupffer cells, play a major role in the removal of opsonized particles [17]. Soluble LPS injected i.p., might not become opsonized since it is not a particulate and therefore may be preferentially processed in the spleen rather than liver. This may account, at least in part, for the increased cytokine and COX-2 gene expression observed in the spleen when compared to the liver.

Specific cell populations of spleen and liver are likely to contribute to observed cytokine expression. Hepatocytes, the parenchyma cells of the liver, account for 60% of total liver cells and 80% of the liver's volume [18]. The primary functions of these cells are exocrine and metabolic in nature. Although they are capable of functioning as antigen-presenting cells in certain situations, they are not primary mediators of immune regulation in the liver [19]. Other, nonparenchymal cells of the liver include Kupffer cells, the resident macrophage, and interstitial dendritic cell types. Both Kupffer and dendritic cells are capable of producing proinflammatory cytokines. The macrophage population of liver (10%) is more than three times larger than the spleen (3%) [20]. However, the opposite is observed with respect to dendritic cell populations. In the spleen, the dendritic cell population is approximately ten times larger compared to liver [20]. It is possible that dendritic cells, which are constitutively activated, might respond more readily to antigen exposure than macrophage cell populations, and as a result, express proinflammatory cytokines to a greater extent [21]. These and other cell types including endothelial and epithelial cells might also contribute differences in spleen and liver mRNA expression. Future examination of macrophage responses at other tissue sites and responses of other cell types is clearly warranted.

Parthenolide's in vitro effects on mediators of inflammation including cytokines (TNF-α, IL-1β and IL-6) [5,13], chemokine (IL-8) [15] and lipid mediators (prostaglandins [6,7], COX [4,5] and leukotrienes [4]) have been extensively studied. Recent research has focused on the role of transcription factor NF-κB [8,10-12]. Notably, transcriptional regulation of cytokine genes including TNF-α [22], IL-6 and IL-8 [23] has been strongly linked to NF-κB activation. Interestingly, parthenolide has been shown to inhibit expression of each of these cytokines [5,13], as well as activation of NF-κB [8,10-12] in cell culture studies. Parthenolide appears to inhibit NF-κB by targeting the IκB (inhibitor of NF-κB) kinase complex [9] which might inhibit proinflammatory cytokine and chemokine gene expression. Relative to other transcription factors like CCAAT/enhancer binding protein (C/EBP)β, NF-κB plays a dominant role in regulation of IL-6 expression in other models of inflammation [24].

In contrast to the observed effects of LPS and parthenolide co-treatment on IL-6 production and gene expression, no inhibitory effect was observed for TNF-α. Although NF-κB has been implicated in the transcriptional regulation of TNF-α, the functional concert of NF-κB with other transcription factors such as activator protein (AP)-1 [25] and C/EBPβ (reviewed by [26]) may override the importance of NF-κB in LPS-induced TNF-α expression. The dual pathway of NF-κB and AP-1 has been shown to enhance production of some proinflammatory cytokines, notably TNF-α [27]. Parthenolide inhibits NF-κB, but has no effect on AP-1 [11]. Therefore, the expression of TNF-α may be compensated for by transcriptional activation by AP-1.

Similar to the effects observed for TNF-α mRNA expression, there were no significant changes in COX-2 mRNA expression of LPS versus LPS plus parthenolide co-treated animals. Hwang et al. [5] demonstrated the inhibitory effects of parthenolide on LPS-induced COX-2 protein and mRNA, however, those studies employed cultured alveolar macrophage cells rather than an in vivo model as described here. No other studies have directly evaluated the effect of parthenolide on COX-2 mRNA. The COX-2 gene is regulated by a number of transcription factors including NF-κB, C/EBPβ and AP-1 as well as cAMP response element-binding protein (CREB) and others. Site-directed mutagenesis studies of basal COX-2 expression in murine lung tumor derived cell lines highlight the role of C/EBPβ and CREB as major transcriptional regulators of COX-2 [28], whereas NF-κB appeared to have no role in COX-2 transcriptional regulation using this model. Thus the lack of inhibitory effect on COX-2 mRNA expression might be explained, in part, by the limited role of NF-κB in COX-2 transcriptional regulation.

IL-1β mRNA expression followed a different pattern than the other two cytokines. IL-6 was decreased and TNF-α was unchanged, while IL-1β levels were increased following co-treatment with LPS and parthenolide. Although IL-1β is also transcriptionally regulated by NF-κB [22,25] and C/EBPβ (reviewed by [29]), similar to IL-6 and TNF-α, it might be differentially regulated in response to LPS. In support of this hypothesis, in vivo studies by Zhou et al. [30] show that mRNA levels of IL-1β in the spleen are not affected under conditions of LPS tolerance whereas both TNF-α and IL-6 are reduced.

## Conclusion

In summary, parthenolide selectively modulated proinflammatory cytokine gene expression in vivo. Only one gene, IL-6, was modestly suppressed which contrasts with many in vitro studies suggesting anti-inflammatory effects of this compound. It is possible that the differences in metabolism and/or distribution of parthenolide could explain contrasting in vivo and in vitro results. LPS also exerted greater effects in spleen than liver on expression of proinflammatory genes. Higher doses of parthenolide might have greater effects on IL-6 but there are solubility issues relative to delivery. In addition, the physiological significance of greater doses would be questionable. Further study of the effects of parthenolide and other herbal constituents on inflammatory gene expression using model animal systems as described here are critical to evaluating efficacy of such supplements as well as elucidating their mechanisms of action.

## List of abbreviations

alpha, α; hour, h; LPS, lipopolysaccharide; TNF, tumor necrosis factor; IL, interleukin; COX, cyclooxygenase; PG, prostaglandin; DMSO, dimethyl sulfoxide; CREB, cAMP response element-binding protein; C/EBPβ, CCAAT/enhancer binding protein beta; NF-κB, nuclear factor kappa B

## Competing interests

The author(s) declare that they have no competing interests.

## Authors' contributions

ATS participated in study design, carried out experiments, performed data analysis and drafted the manuscript. JJP participated in study design and coordination, as well as editing and revision of the final manuscript.

## References

[B1] Groenewegen WA, Heptinstall S (1990). A comparison of the effects of an extract of feverfew and parthenolide, a component of feverfew, on human platelet activity in-vitro. J Pharm Pharmacol.

[B2] Schinella GR, Giner RM, Recio MC, Mordujovich dB, Rios JL, Manez S (1998). Anti-inflammatory effects of South American Tanacetum vulgare. J Pharm Pharmacol.

[B3] Jain NK, Kulkarni SK (1999). Antinociceptive and anti-inflammatory effects of Tanacetum parthenium L. extract in mice and rats. J Ethnopharmacol.

[B4] Sumner H, Salan U, Knight DW, Hoult JR (1992). Inhibition of 5-lipoxygenase and cyclooxygenase in leukocytes by feverfew. Involvement of sesquiterpene lactones and other components. Biochem Pharmacol.

[B5] Hwang D, Fischer NH, Jang BC, Tak H, Kim JK, Lee W (1996). Inhibition of the expression of inducible cyclooxygenase and proinflammatory cytokines by sesquiterpene lactones in macrophages correlates with the inhibition of MAP kinases. Biochem Biophys Res Commun.

[B6] O'Neill LA, Barrett ML, Lewis GP (1987). Extracts of feverfew inhibit mitogen-induced human peripheral blood mononuclear cell proliferation and cytokine mediated responses: a cytotoxic effect. Br J Clin Pharmacol.

[B7] Pugh WJ, Sambo K (1988). Prostaglandin synthetase inhibitors in feverfew. J Pharm Pharmacol.

[B8] Uchi H, Arrighi JF, Aubry JP, Furue M, Hauser C (2002). The sesquiterpene lactone parthenolide inhibits LPS- but not TNF-alpha-induced maturation of human monocyte-derived dendritic cells by inhibition of the p38 mitogen-activated protein kinase pathway. J Allergy Clin Immunol.

[B9] Hehner SP, Hofmann TG, Droge W, Schmitz ML (1999). The antiinflammatory sesquiterpene lactone parthenolide inhibits NF- kappa B by targeting the I kappa B kinase complex. J Immunol.

[B10] Hehner SP, Heinrich M, Bork PM, Vogt M, Ratter F, Lehmann V (1998). Sesquiterpene lactones specifically inhibit activation of NF-kappa B by preventing the degradation of I kappa B-alpha and I kappa B-beta. J Biol Chem.

[B11] Bork PM, Schmitz ML, Kuhnt M, Escher C, Heinrich M (1997). Sesquiterpene lactone containing Mexican Indian medicinal plants and pure sesquiterpene lactones as potent inhibitors of transcription factor NF-kappaB. FEBS Lett.

[B12] Rungeler P, Castro V, Mora G, Goren N, Vichnewski W, Pahl HL (1999). Inhibition of transcription factor NF-kappaB by sesquiterpene lactones: a proposed molecular mechanism of action. Bioorg Med Chem.

[B13] Smolinski AT, Pestka JJ (2005). Modulation of proinflammatory cytokine production in vitro and in vivo by the herbal constituents apigenin (chamomile), ginsenoside Rb_1 _(ginseng) and parthenolide (feverfew). Food Chem Toxicol.

[B14] Kang BY, Chung SW, Kim TS (2001). Inhibition of interleukin-12 production in lipopolysaccharide-activated mouse macrophages by parthenolide, a predominant sesquiterpene lactone in Tanacetum parthenium: involvement of nuclear factor-kappaB. Immunol Lett.

[B15] Mazor RL, Menendez IY, Ryan MA, Fiedler MA, Wong HR (2000). Sesquiterpene lactones are potent inhibitors of interleukin 8 gene expression in cultured human respiratory epithelium. Cytokine.

[B16] Howard HL, McLaughlin-Taylor E, Hill RL (1990). The effect of mouse euthanasia technique on subsequent lymphocyte proliferation and cell mediated lympholysis assays. Lab Anim Sci.

[B17] Schuurman H, Krajnc-Franken M, Kuper C, van Loveren H, Vos J, Haschek W, Rousseaux C (1998). Immune System. Fundamentals of Toxicologic Pathology.

[B18] Popp J, Cattely R, Haschek W, Rousseaux C (1998). Hepatobiliary System. Fundamentals of Toxicologic Pathology.

[B19] Lau AH, Thomson AW (2003). Dendritic cells and immune regulation in the liver. Gut.

[B20] Zhang Y, Shlomchik WD, Joe G, Louboutin JP, Zhu J, Rivera A (2002). APCs in the liver and spleen recruit activated allogeneic CD8+ T cells to elicit hepatic graft-versus-host disease. J Immunol.

[B21] Banchereau J, Steinman RM (1998). Dendritic cells and the control of immunity. Nature.

[B22] Mercurio F, Manning AM (1999). Multiple signals converging on NF-kappaB. Curr Opin Cell Biol.

[B23] Baldwin AS (1996). The NF-kappa B and I kappa B proteins: new discoveries and insights. Annu Rev Immunol.

[B24] Baeuerle PA, Baichwal VR (1997). NF-kappa B as a frequent target for immunosuppressive and anti-inflammatory molecules. Adv Immunol.

[B25] Tak PP, Firestein GS (2001). NF-kappaB: a key role in inflammatory diseases. J Clin Invest.

[B26] Poli V (1998). The role of C/EBP isoforms in the control of inflammatory and native immunity functions. J Biol Chem.

[B27] Yokoo T, Kitamura M (1996). Dual regulation of IL-1 beta-mediated matrix metalloproteinase-9 expression in mesangial cells by NF-kappa B and AP-1. Am J Physiol.

[B28] Wardlaw SA, Zhang N, Belinsky SA (2002). Transcriptional regulation of basal cyclooxygenase-2 expression in murine lung tumor-derived cell lines by CCAAT/enhancer-binding protein and activating transcription factor/cAMP response element-binding protein. Mol Pharmacol.

[B29] Wedel A, Ziegler-Heitbrock HW (1995). The C/EBP family of transcription factors. Immunobiology.

[B30] Zhou HR, Islam Z, Pestka JJ (2003). Kinetics of lipopolysaccharide-induced transcription factor activation/inactivation and relation to proinflammatory gene expression in the murine spleen. Toxicol Appl Pharmacol.

